# Characterization and Biocompatibility of Biopolyester Nanofibers

**DOI:** 10.3390/ma2041520

**Published:** 2009-10-09

**Authors:** Daisuke Ishii, Tang Hui Ying, Tetsuji Yamaoka, Tadahisa Iwata

**Affiliations:** 1 Department of Materials Chemistry, Faculty of Science and Engineering, Ryukoku University / 1-5 Yokotani, Seta Oe-cho, Otsu-shi, Shiga 520-2194, Japan; E-Mail: ishiidai@rins.ryukoku.ac.jp (D.I.); 2 Bioengineering Laboratory, RIKEN Institute/Hirosawa 2-1, Wako-shi, Saitama 351-0198, Japan; E-Mail: nicole_thy@yahoo.com (T.H.Y.); 3 Universiti Sains Malaysia/11800 Penang, Malaysia; 4 Department of Biomedical Engineering, Advanced Medical Engineering Center, National Cardiovascular Center Research Institute, 5-7-1 Fujishirodai, Suita-shi, Osaka 565-8565, Japan; E-Mail: yamtet@ri.ncvc.go.jp (T.Y.); 5 Department of Biomaterial Sciences, Graduate School of Agricultural and Life Sciences, The University of Tokyo/1-1-1 Yayoi, Bunkyo-ku, Tokyo 113-8657, Japan

**Keywords:** poly(hydroxyalkanoate), poly(lactide), nanofibers, biocompatibility

## Abstract

Biodegradable nanofibers are expected to be promising scaffold materials for biomedical engineering, however, biomedical applications require control of the degradation behavior and tissue response of nanofiber scaffolds *in vivo*. For this purpose, electrospun nanofibers of poly(hydroxyalkanoate)s (PHAs) and poly(lactide)s (PLAs) were subjected to degradation tests *in vitro* and *in vivo*. In this review, characterization and biocompatibility of nanofibers derived from PHAs and PLAs are described. In particular, the effects of the crystalline structure of poly[(*R*)-3-hydroxybutyrate], stereocomplex structure of PLA, and monomer composition of PHA on the degradation behaviors are described in detail. These studies show the potential of biodegradable polyester nanofibers as scaffold material, for which suitable degradation rate and regulated interaction with surrounding tissues are required.

## 1. General Introduction

### 1.1. Biodegradable polymers for biomedical applications

In the field of medical sciences, tissue engineering has been extensively studied to overcome the problems of conventional methods such as organ transplantation and usage of artificial organs [1]. In tissue engineering, the proliferation and differentiation of cultured cells for deficiency repair has to be artificially controlled. The development of scaffold materials on which cells proliferate and differentiate has been a major concern in tissue engineering. A major objective of the development of scaffold materials is to mimic the structure and function of extracellular matrix in a living system. The extracellular matrix is composed of fibrous network made from collagens. It not only mechanically supports cells in the tissues but also affects their functions through so-called “cell-extracellular matrix interaction” [2]. The growth, organization, differentiation, and even the death of cells are regulated by the interactions with the extracellular matrix [3].

Conventionally, collagens and gelatins extracted from animals have been used to produce scaffolds. However, these native collagens contain considerable amount of endotoxins and other peptides that can stimulate tissue reactions in the human body. Therefore, the removal of these contaminants is crucial for medical applications. Furthermore, the usage of collagens from cows is strictly limited for fear of spreading Bovine Spongiform Encephalopathy (BSE) as has been known to occur with collagens extracted from some of the infected tissues such as bone marrow and eyeballs. Hence, the development of scaffold material alternatives to collagens and gelatins has been attempted [4].

Some polymeric materials, both from natural and synthetic origin, degrade under physiological conditions. For example, poly(glycolic) acid has been utilized in bioabsorptive sutures without the need for removal after healing of wounds. Such biomaterials require a suitable degree of biodegradability and biocompatibility depending on their purposes. In order to regulate these properties, polymeric biomaterials with diverse monomeric compositions, polymeric mixture ratios, or morphologies has been prepared and tested for their biodegradation behaviors *in vivo* or *in vitro*.

**Figure 1:**
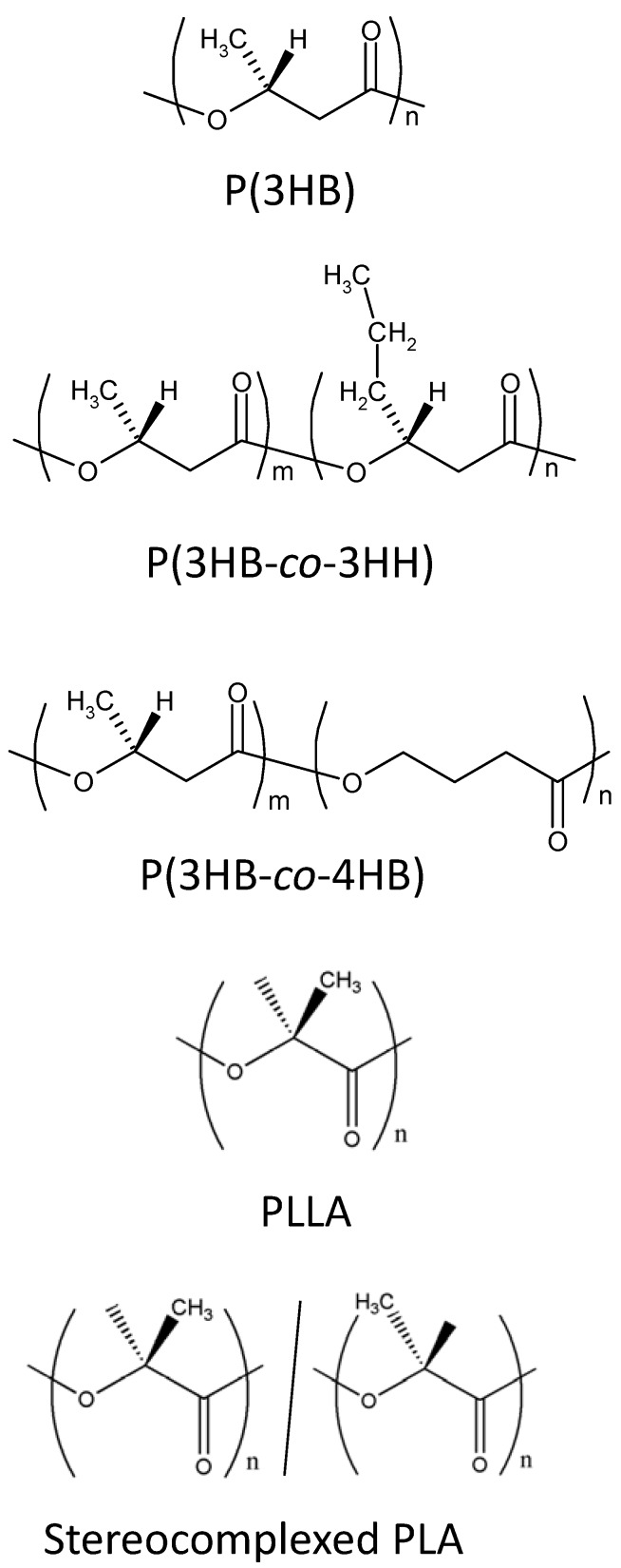
Chemical structures of PHAs, PLLA and stereocomplexed PLA.

Poly(hydroxyalcanoate)s (PHAs) and poly(lactide) (PLA) have been attracting the largest attention as biodegradable and biocompatible polymers for medical applications [5]. PHAs are produced by many species of microorganisms as their intracellular energy and carbon storage substance [6]. Since the first report of poly[(*R*)-3-hydroxybutyrate] [P(3HB)] by Lemoigne in 1920s [7], a wide variety of PHAs, such as poly[(*R*)-3-hydroxybutyrate-*co*-(*R*)-3-hydroxyvalerate] [P(3HB-*co*-3HV)], poly[(*R*)-3-hydroxybutyrate-*co*-(*R*)-3-hydroxyhexanoate] [P(3HB-*co*-3HH)], poly[(*R*)-3-hydroxybutyrate-*co*-4-hydroxybutyrate] [P(3HB-*co*-4HB)], and poly(4-hydroxybutyrate) [P(4HB)] have been found or developed by controlling the cultivation conditions of microorganisms [6,8]. The chemical structures of these PHA copolymers are shown in Figure 1. They show varying mechanical properties. For example, P(3HB) homopolymer has high tenacity and an elastic modulus as high as more than 1 GPa [9]. On the other hand, P(4HB) have high flexibility, showing 1,000% elongation to break [10]. Furthermore, PHAs show low degrees of cytotoxicity in Nature, because the monomeric components of PHAs are often found in human organs as metabolic products [11]. Such versatility and safety enable PHAs for application as medical biomaterials, such as surgical sutures and wound dressings [12].

Poly(lactide) (PLA) is one of a few polymers that is used practically in various medical materials such as implants and sutures [13]. PLA possesses mechanical properties sufficient to endure the mechanical loads applied in the human body. However, it is readily hydrolyzed both under enzymatic and non-enzymatic conditions [14]. The high susceptibility of PLA towards hydrolysis becomes a shortcoming when the long-time storage under physiological conditions is required. Various efforts to overcome this shortcoming have been attempted. One of such efforts is the formation of stereocomplexes in PLA materials. Stereocomplexed PLA is a characteristic crystalline form of PLA [15,16]. Sterically stable racemic crystals of stereocomplexed PLA are formed by complexing poly(l-lactide) (PLLA) and poly(d-lactide) (PDLA) that adopt left-handed and right-handed helix molecular conformations, respectively. [17] As a result, stereocomplexed PLA has a melting temperature of 230 °C that is 50 °C higher than PLLA and PDLA. [15] Furthermore, it has been reported that stereocomplexed PLA is more stable against hydrolysis than PLLA. [18,19,20] This finding offers the possibility for controlling the hydrolytic behavior of PLA material by the formation of stereocomplexes.

### 1.2. Preparation of nanofibers of biodegradable polymers by electrospinning

Recently, the formation of nanofibers with diameters ranging from several tens to hundreds of nanometers has been extensively studied as a novel method for producing scaffolds [21,22,23]. In particular, electrospinning is prevailing as the most convenient method for the fabrication of polymeric nanofibers. In the electrospinning process, the polymer solution is extruded from a nozzle to which a high electric voltage is applied [24]. The extruded polymer solution is scattered by the repulsion of electrical charges accumulated at the surface of the solution. Then the droplets of the solution are elongated by the electrostatic force operating between droplet and substrate. The nanofiber is formed by the rapid evaporation of solvent from the droplet. Nanofiber scaffolds, formed by the accumulation of nanofibers, have fine pores and grooves as small as a few micrometers wide. Such fine structural features facilitate the adhesion and proliferation of cells. It is necessary for nanofiber scaffolds to sustain sufficient strength to support regenerating tissue cells and to be degraded after the tissue regeneration is completed. To meet these demands, various kinds of biodegradable and biocompatible polymers have been processed into nanofibers. Furthermore, the fiber morphology, crystalline structure, and degradation behavior of the nanofibers have been investigated. [25,26,27] Although the fabrication of nanofibers of PHAs and PLAs has also been reported [26,28,29,30,31,32,33], information on the relation between the fine structure and degradation behavior of nanofiber has not yet been obtained. In particular, *in vivo* studies on the biocompatibility and degradation behavior of PHA and PLA nanofibers have yet to be performed.

## 2. Preparation and Characterization of PHA and PLA Nanofibers

### 2.1. PHA nanofibers

**Figure 2:**
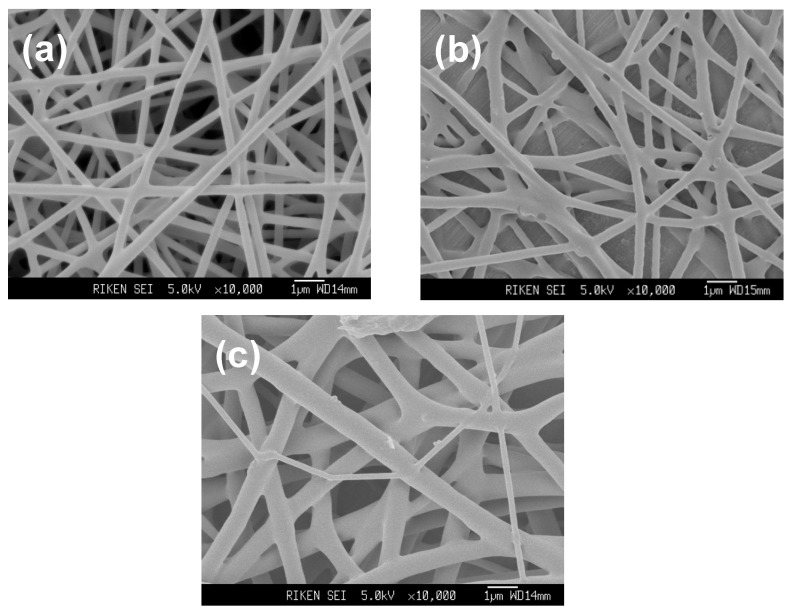
SEM images of P(3HB) nanofiber electrospun from HFIP solution with a polymer concentration of (**a**) 0.5 wt%, (**b**) 1 wt% and (**c**) 2.5 wt% [52].

**Figure 3:**
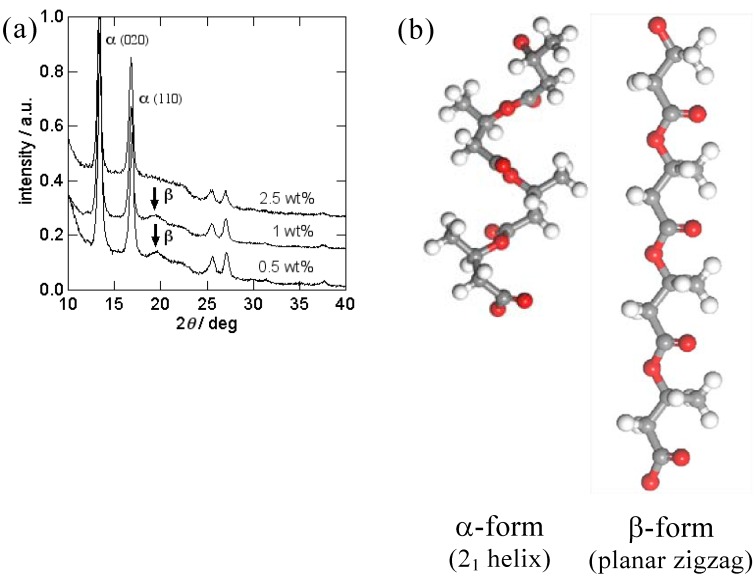
**(a)** WAXD profiles of as-spun P(3HB) nanofiber mats and (b) molecular conformations of α-and β-form of P(3HB) [52].

P(3HB) nanofibers were electrospun from 1,1,1,3,3,3-hexafluoro-2-propanol (HFIP) solution with a polymer concentration ranging from 0.5 to 2.5 wt%. Electospinning was performed on an Esprayer ES-2000 electrospinning device (Fuence, Co. Ltd.). The P(3HB) solutions were extruded with a speed of 1.4 mL·h^−1^ from a syringe needle with an inner diameter of 0.5 mm. Electrical voltage of 15 kV was applied to the syringe. SEM images of the P(3HB) nanofibers are shown in Figure 2. While the nanofibers spun from 2.5 wt% solution had the average diameter of 560 nm, nanofibers spun from 1wt% and 0.5 wt% solutions had the average diameters of 350 nm and 280 nm, respectively. Figure 3 shows the WAXD profiles of as-spun P(3HB) nanofibers. In all the profiles, diffraction peaks assigned to the α-form crystal with 2_1_ helix molecular conformation were observed. Furthermore, in the WAXD profiles of 0.5wt% and 1wt% nanofibers, a diffraction peak assigned to β-form structure with planar zigzag conformation was observed at 2θ = 19.6° [34]. This shows that β-form as well as α-form crystals are formed in the 0.5 wt% and 1 wt% nanofibers. Considering that the nanofibers spun from 2.5 wt% solution did not show β-form diffraction, the electrospinning from the lower polymer concentrations causes the greater stretching of molecular chains favoring the formation of β-form structure.

**Figure 4:**
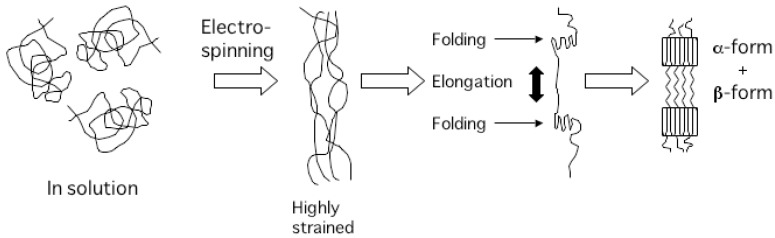
Schematic representation of the formation mechanism of β-form structure in P(3HB) nanofiber [52].

The formation of β-form structure was first reported by Orts *et al*. in the cold-stretched poly[3HB-*co*-3HV] films [34]. Subsequently, the β-form generation was reported for the materials of P(3HB) and its copolymers processed in various ways, including hot-stretched films [35], cold-stretched films [36], cold-drawn/two-step-drawn fibers [9,37], and one-step drawn fibers after isothermal crystallization [38]. From these studies, it was found that the β-form structure is generated from the amorphous chains between the lamella crystals [39]. Because high strain is applied to the polymer chains by electrospinning process, the polymer chains between lamella crystals are strongly elongated to form β-form conformation when polymer chains are folded to form lamella crystal. The schematic representation of the formation of β-form structure is shown in Figure 4.

It is known that P(3HB) materials with β-form structure has a higher mechanical strength, elasticity and biodegradability than those with only α-form structure [9,34,35,36,37,38]. Therefore, it is expected that the formation of β-form structure in the P(3HB) nanofiber leads to the development of scaffold materials with improved mechanical properties and biodegradability.

Nanofibers of PHA copolymers, electrospun from 1wt% solution in HFIP in a similar way to P(3HB) nanofiber, showed different fiber morphologies depending on monomer composition. The width decreased in the order of P(3HB) ~ P(3HB-*co*-5mol%-3HH) (520 nm) > P(3HB-*co*-97mol%-4HB) (220 nm) > P(3HB-*co*-7mol%-4HB) (190 nm). It has been reported that the width increases proportionally with the molecular weight of the polymer [40]. This is because higher degree of chain entanglement due to high molecular weight is assumed to make it harder for the electrostatic forces to pull, or extend individual molecular chains [41]. Accordingly, the matrices of the P(3HB) and P(3HB-*co*-5mol%-3HH) nanofibers consisted of larger fibers compared to the P(3HB-*co*-97mol%-4HB) nanofibers because of their high molecular weight (Table 1). Interestingly, only the P(3HB-*co*-7mol%-4HB) formed nanofibers with irregular shapes with intermittent spindle-like beads on string (images not shown). Possibly the formation of the beaded P(3HB-*co*-7mol%-4HB) nanofibers is the result of low net charge density. Previous studies have shown that higher net charge density favors the formation of bead-free fibers [42,43]. According to Ref. [43], the net charge density is inversely proportional to the mass of dry polymer (i.e., mass of scaffolds collected from electrospinning), if the other experimental conditions such as jet current, collecting time and polymer concentration are the same. The net charge density decreased in the order of P(3HB-*co*-5mol%-3HH) (1058C/l) > P(3HB) (1002 C/l) > P(3HB-*co*-97mol%-4HB) (778C/l) > P(3HB-*co*-7mol%-4HB) (484 C/l). In this study, the P(3HB-*co*-7mol%-4HB) nanofibers had the highest collected mass.

Table 1 summarizes the mechanical properties of the obtained electrospun PHA scaffolds. The mechanical properties of all the as-spun scaffolds were comparable to those of human skin, and hence suggest they are mechanically stable in supporting regenerated tissues. The Young’s modulus of the as-spun scaffolds increased in the order of P(3HB-*co*-97mol%-4HB) << P(3HB-*co*-7mol%-4HB) < P(3HB) < P(3HB-*co*-5mol%-3HH). Low Young’s modulus, that is, high ductility is a characteristic property in rubber-state amorphous polymers. Accordingly, this indicates that the P(3HB-*co*-97mol%-4HB) fibers are more amorphous than the other scaffolds, and this is consistent with the WAXD results (data not shown). The distinct mechanical properties of the PHA could find different use as scaffolds for tissue engineering. For example, the 3HB-rich scaffolds which are more rigid could serve as preferential substrates for directional cell migration while the compliant 4HB-rich scaffolds could be used to promote cell motility. The EtO sterilization and the immersion in PBS buffer little affected the mechanical properties of all the scaffolds.

**Table 1:** Mechanical properties of PHA copolymers before and after *in vivo* implantation and *in vitro* degradation [54].

Material	Condition	Mechanical properties	
		Tensile strength (MPa)	Young’s Modulus (MPa)
P(3HB)	As-spun	17	223
	Sterilized	15	234
	4 weeks *in vivo* *in vitro*	12 14	182 220
	12 weeks *in vivo* *in vitro*	15 13	152 194
P(3HB-*co*-5mol%-3HH)	As-spun	15	277
	Sterilized	12	272
	4 weeks *in vivo* *in vitro*	12 13	268 208
	12 weeks *in vivo* *in vitro*	ND ^b^ 15	ND ^b^ 230
P(3HB*-co*-7mol%-4HB)	As-spun	8	184
	Sterilized	8	139
	4 weeks *in vivo* *in vitro*	ND ^b^ 8	ND ^b^ 163
	12 weeks *in vivo* *in vitro*	ND ^b^ 9	ND ^b^ 110
P(3HB*-co*-97mol%-4HB)	As-spun	13	9
	Sterilized	15	16
	4 week*s in vivo* *in vitro*	4 11	12 14
	12 weeks *in vivo* *in vitro*	ND ^b^ 14	ND ^b^ 16
Skin^a^		5-30	15-150

^a^ Cited from Zong, X. *et al.*, *Biomaterials* 2005, *26*, 5330-5338 [56].

^b^ Not determined.

### 2.2. PLA nanofibers

PLLA (*M*_w_ = 6.2 × 10^5^, *M*_w_/*M*_n_ = 2.1, Polysciences, Inc.) and PDLA (*M*_w_ = 3.3 × 10^5^, *M*_w_/*M*_n_ = 1.5, kindly supplied by Prof. Won-ki-Lee, Pukyong Univ., South Korea) were dissolved separately in HFIP with the polymer concentration of 1 wt%. Stereocomplexed PLA nanofiber was electrospun from 1:1 mixture of the PLLA and PDLA solutions. PLLA and PDLA nanofiber was electrospun from the PLLA and PDLA solutions, respectively. PLLA, PDLA, and stereocomplexed PLA nanofibers were then annealed in an oven at 100 °C for 8 h. All nanofiber samples were stored at room temperature until use.

**Figure 5:**
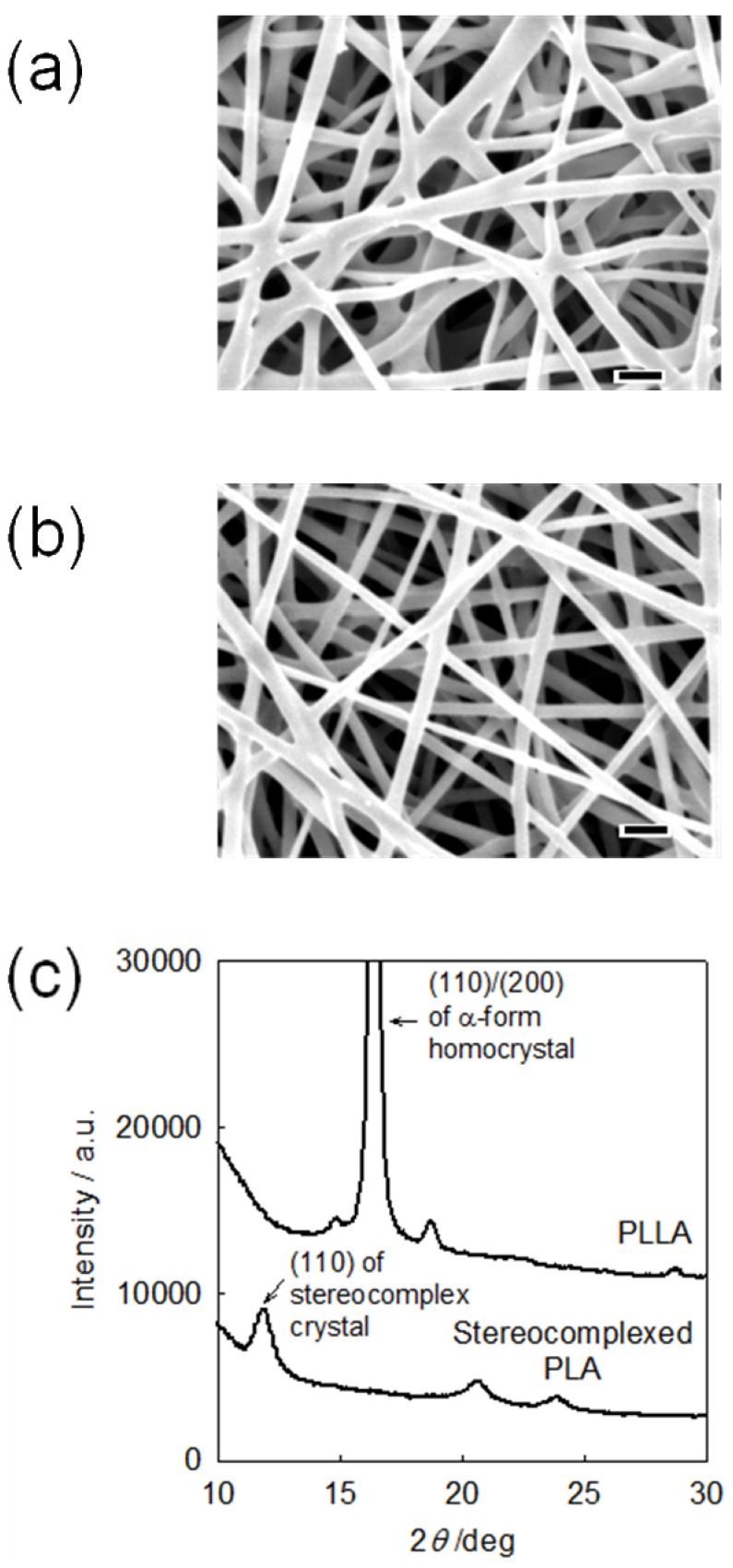
**(a)** SEM image of PLLA nanofiber, **(b)** SEM image of stereocomplexed PLA nanofiber, and **(c)** WAXD profiles of PLA nanofibers [52].

Figure 5(a) and (b) shows the SEM images of the PLLA and stereocomplexed PLA nanofibers, respectively. Both nanofibers possess similar morphology with the average fiber diameter of about 300 nm. However, totally different crystalline structure is formed in these nanofibers, as seen from the WAXD profiles in Figure 5(c). PLLA nanofibers showed diffraction peaks at 2*θ* = 15.1°, 16.5° [assigned to (110)/(200)], and 18.1° that are assigned to α-form homocrystal of PLA [17]. On the other hand, stereocomplexed PLA nanofibers showed diffraction peaks at 2*θ* = 12.0° [assigned to (110)], 20.8°, and 24.1° that are assigned only to the stereocomplex crystal of PLA. [17] This shows that the stereocomplexed PLA nanofibers consist of only the stereocomplex crystal and does not contain homocrystals of PLLA and PDLA at all.

Until now, the formation of stereocomplex fibers by solution spinning [44] or melt spinning [45,46] from the mixed solution or melt of PLLA and PDLA has been attempted. However, homocrystals as well as stereocomplex crystals were formed, even after long-time annealing at elevated temperatures as high as 180 °C and repeated stretching. This is possibly because the alignment of the molecular chains of PLLA and PDLA is not well developed. Tsuji and his coworkers also performed electrospinning to prepare stereocomplexed PLA nanofibers [47]. However, their nanofibers contained considerable amount of homopolymer crystal. In our case [52], the polymer chains are forced to align due to the application of electrical forces and the rapid solidification. This may help the development of side-by-side alignment of PLLA and PDLA chains that is required to form stereocomplex crystal. Therefore, it is supposed that the association of PLLA and PDLA occurs in the as-spun nanofiber, even though no crystalline order is developed. Subsequently, annealing above the crystallization temperature causes the arrangement of molecular chains and the formation of stereocomplex crystal. The formation of racemic crystal was performed by annealing the as-spun nanofibers at 100 °C, that is 80 °C lower than used in previously reported studies.

## 3. Biocompatibility of PHA Nanofibers

### 3.1. Enzymatic degradation of P(3HB) nanofibers

**Figure 6:**
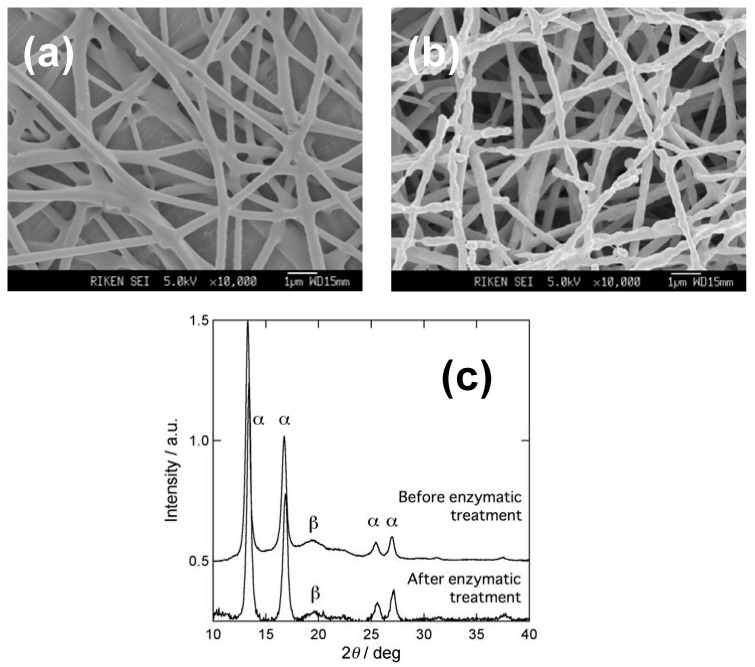
P(3HB) nanofiber before and after enzymatic treatment by PHB depolymerase from *Ralstonia pickettii* T1. **(a)** SEM image before enzymatic treatment, **(b)** SEM image after enzymatic treatment, and **(c)** WAXD profiles before and after enzymatic treatment [52].

**Figure 7:**
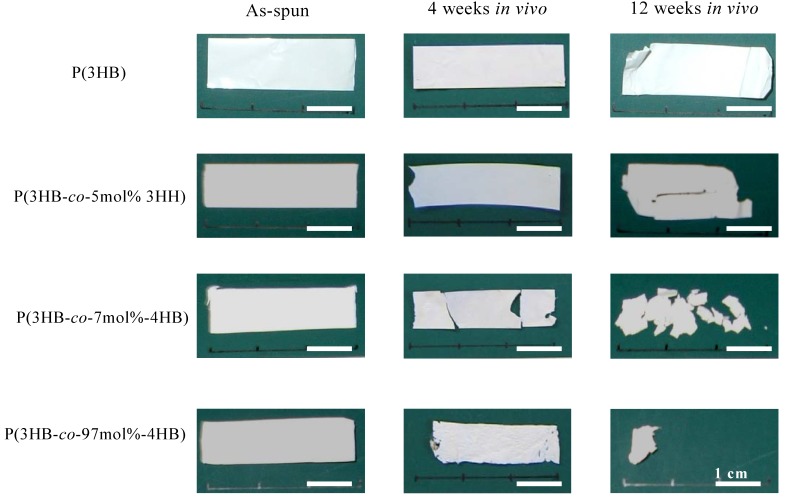
PHA nanofiber mats after subcutaneous implantation in rat for 4 to 12 weeks [54].

P(3HB) nanofiber mat with a size of 3 mm × 20 mm was immersed in a 2 mL solution of 0.5 M sodium phosphate buffer (pH 7.4) at 4 °C. A 5 μL of 200 μg·mL^−1^ solution of extracellular PHB depolymerase purified from *Ralstonia pickettii* T1 was added to the buffer. The concentration of enzyme in the mixture solution was about 1 μg/mL. Then the solution was kept to stand at 37 °C for 0.5 h, 1 h, or 1.5 h for the enzymatic hydrolysis. Figure 6 (a) and (b) show SEM images of P(3HB) nanofibers, before and after enzymatic treatment, respectively. In contrast to the smooth surface of nanofibers before enzymatic treatment, nanofibers after enzymatic treatment showed an irregular surface. Figure 7(c) shows the WAXD profiles of the partially hydrolyzed P(3HB) nanofibers spun from 1wt% HFIP solution. While the intensity of (020) diffraction of α-form at 13.4° remained unchanged, the intensity of β-form diffraction at 2θ = 19.6° was decreased. This shows that the P(3HB) molecular chains with β-form structure undergo enzymatic hydrolysis more readily than those with α-form structure. Similar hydrolytic behavior has also been observed in melt-spun P(3HB) fibers [48].

### 3.2. In vivo and in vitro degradation of PHA nanofibers

The nanofiber mats with the size of 1 × 1 cm^2^ and 1 × 3 cm^2^ were implanted subcutaneously at the backbone 12-weeks-old male Wistar rats. The grouping of the rats was based on the duration of observation for 4 and 12 weeks. Upon explantation, the scaffolds measuring 1 × 1 cm^2^ were stored in 2.5% glutaraldehyde solution until further analysis by SEM. The retrieved 1 × 3 cm^2^ scaffolds were treated with 1.25 wt% trypsin to remove the surrounding tissues.

**Figure 8:**
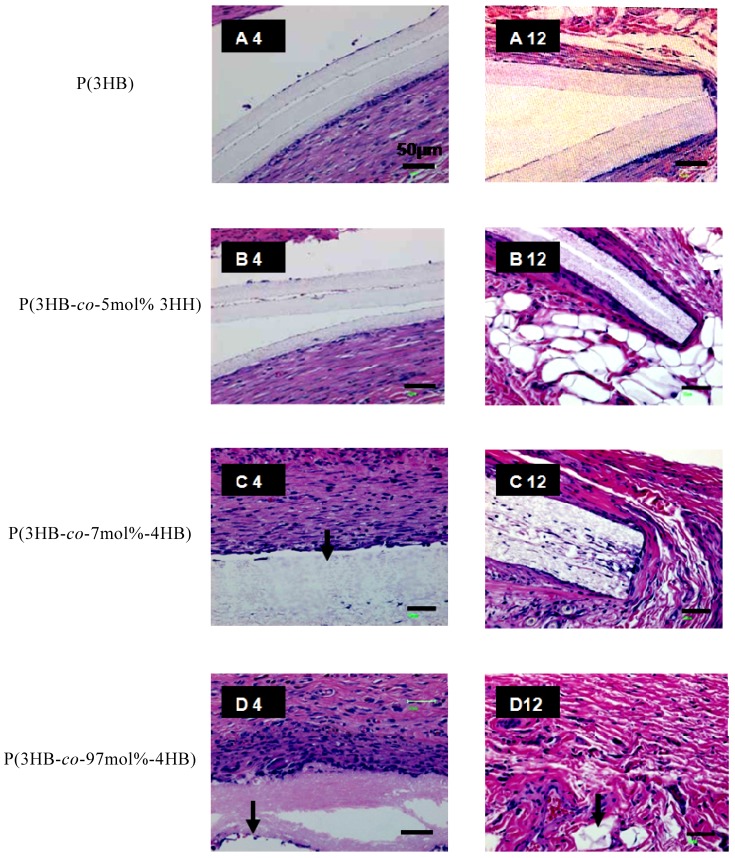
Phase contrast images of hematoxylin-eosin stained ultrathin sections of PHA nanofiber scaffolds after *in vivo* degradation for 4 to 12 weeks. Scale bars = 50 m [54].

PHA nanofiber mats, retrieved after subcutaneous implantion in rats for 4 to 12 weeks, showed various changes in their physical appearance (Figure 7). After 4-weeks implantation, both the P(3HB) and P(3HB-*co*-5mol%-3HH) mats remained in their initial form. In contrast, the P(3HB-*co*-7mol%-4HB) nanofiber mat was fragmented into three large pieces while the P(3HB-*co*-97mol%-4HB) nanofiber mat demonstrated slight shrinkage and thinning of the nanofibrous layers. Significant changes in physical appearance were observed for all three electrospun PHA copolymers explanted after 12-weeks implantation. Only a small piece of the P(3HB-*co*-97mol%-4HB) nanofiber was retrieved, indicating enhanced bioabsorption of this 4HB-rich copolymer. In contrast, the P(3HB-*co*-7mol%-4HB) nanofiber was degraded into small fragments while the P(3HB-*co*-5mol%-3HH) nanofiber displayed crevices on its surface. No changes were observed for P(3HB) nanofiber. The hematoxylin-eosin stained histological sections of the electrospun PHA nanofibers at different period of subcutaneous implantation are shown in Figure 8. Histological observations indicate that all the three copolymer nanofibers elicited fairly mild tissue response relative to that of the P(3HB) nanofiber throughout the course of study. After four weeks of implantation, some parts of the P(3HB-*co*-97mol%-4HB) nanofiber bordering the interface were degraded, as evidenced by the small fragments broken off from the main scaffold (Figure 8 D4). More macrophages were found to be present along the interface connected to this copolymer in comparison to the electrospun P(3HB-*co*-7mol%-4HB) and P(3HB-*co*-5mol%-3HH) (Figure 8 B4 and C4). This phenomenon is desirable during wound healing because the presence of macrophages is necessary for the regeneration of many cell types [49]. The presence of thin connective tissue surrounding the P(3HB-*co*-97mol%-4HB) nanofiber was also observed.

The most promising finding was the tissue response after 12 weeks of implantation for the P(3HB-*co*-97mol%-4HB) nanofiber. No fibrous encapsulation was observed around the degraded copolymer and there was also a substantial drop in the number of inflammatory cells (Figure 8 B12). This observation is similar to a study done on the biocompatibility of P(4HB) implanted subcutaneously in rats by Martin *et al*. [50], that reported minimal inflammatory responses. In this study, the number of inflammatory cells surrounding the electrospun P(3HB-*co*-7mol%-4HB) and P(3HB-*co*-5mol%-3HH) did not appear to have lessen. The muscle cells surrounding these two scaffolds appeared compact as a result of inflammatory reaction (Figure 8 B and C). After 12 weeks of implantation, the number of macrophages bordering the electrospun P(3HB) increased. Inflammation was obvious due to the compacted muscle cells surrounding the scaffold. The difference in tissue response to the electrospun P(3HB-*co*-97mol%-4HB) and the electrospun scaffolds with higher molar fraction of 3HB reflected their distinct physical properties. It has been reported that rigid polymers, such as P(3HB), elicit acute inflammatory reactions because they exert a continuous mechanical stimulus to the surrounding tissues of the implants [51]. Although the tissue response to the electrospun P(3HB-*co*-7mol%-4HB) and electrospun P(3HB-*co*-5mol%-3HH) was slightly more pronounced than that of the electrospun P(3HB-*co*-97mol%-4HB), the overall local tissue response to all three copolymers were found to be mild. The results have confirmed the biocompatibility of all three types of electrospun PHA copolymers.

**Figure 9:**
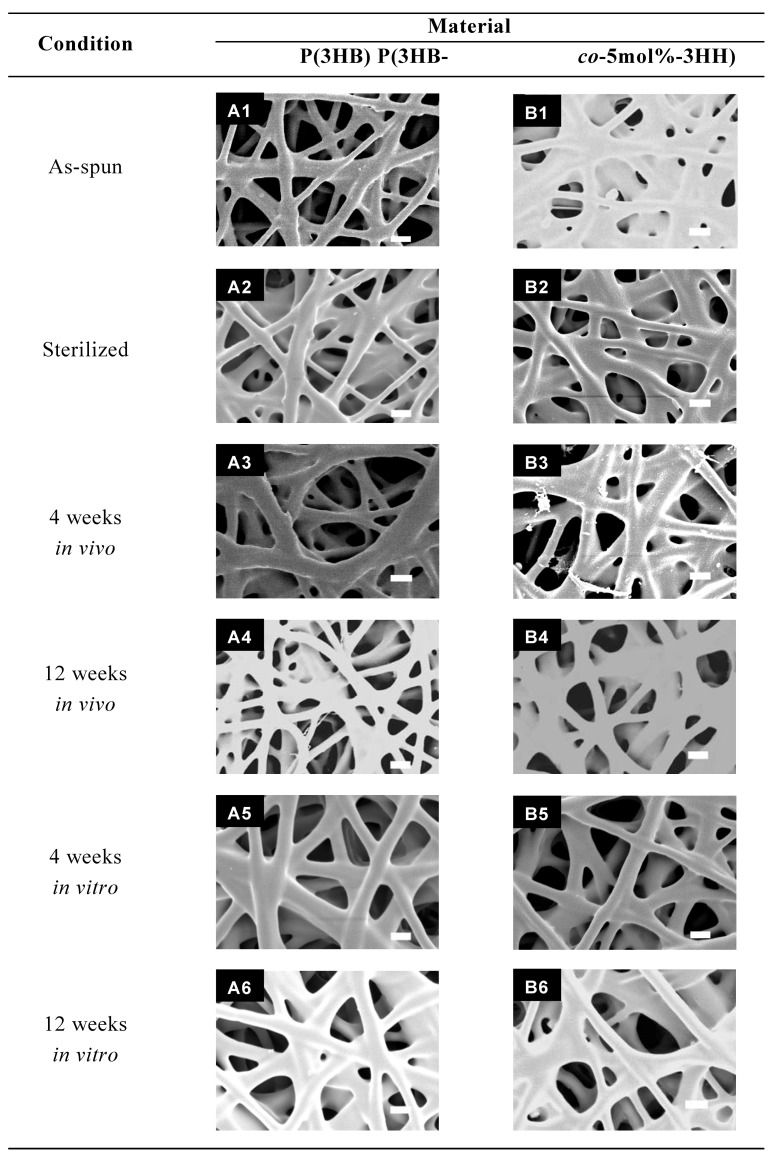

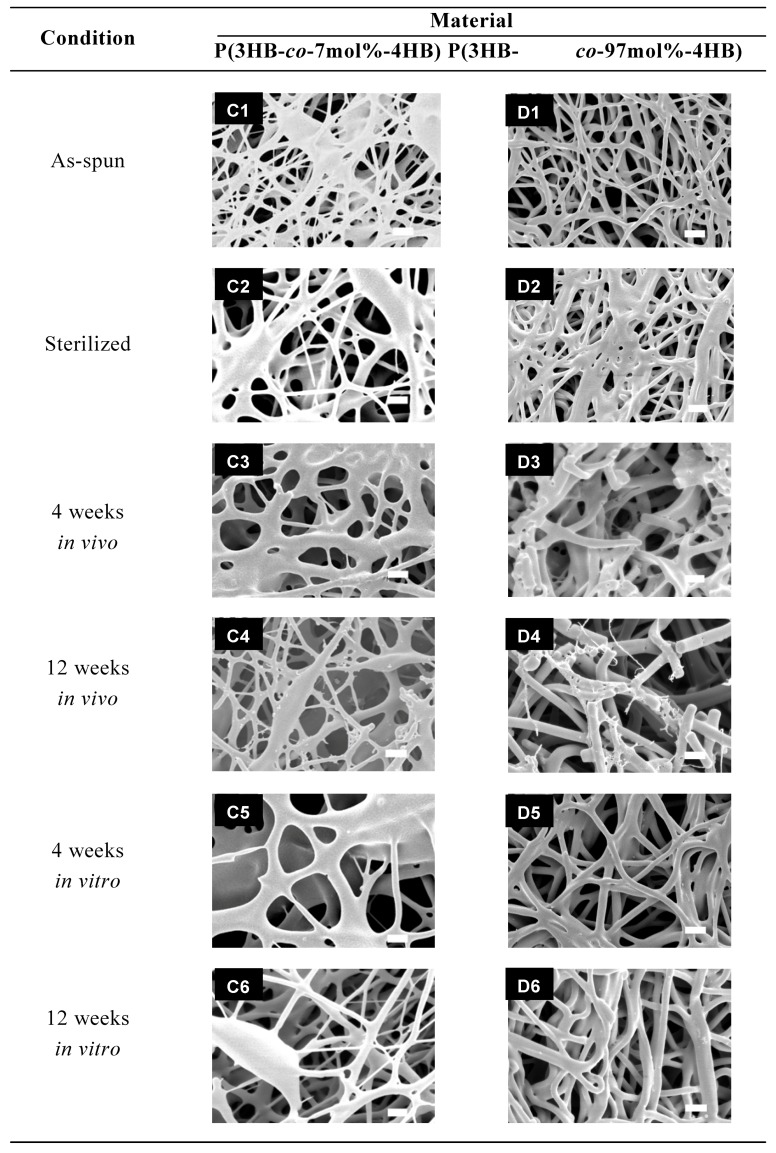
SEM images of PHA nanofiber scaffolds after *in vivo* or *in vitro* degradation for 4 to 12 weeks. Scale bars = 1 μm [54].

The microscopic morphology of the nanofibers after the implantation was observed by SEM. After 4-weeks implantation, the nanofibers of P(3HB), P(3HB*-co*-5mol%-3HH) and P(3HB*-co*-7mol%-4HB) (Figure 9 A3, B3 and C3) maintained their structural integrity. In contrast, the P(3HB*-co*-97mol%-4HB) nanofiber showed fragmentation (Figure 9 D3). After 12-weeks, surface erosion became more evident as the density of the nanofibers decreased remarkably due to fragmentation of the nanofibers to shorter segments. The progression of bioabsorption was also evidenced by the formation of pores on the surface of these nanofibers as indicated by the arrow in Figure 9 D4. After 4-weeks and 12-weeks implantation, the P(3HB*-co*-97mol%-4HB) nanofiber appeared to have swollen (Figure 9 D3 and D4). Interestingly, remnants of the 3-D morphology were still noticeable, despite of the enhanced bioabsorption. Slight decrease in the nanofiber density was also observed for the P(3HB*-co*-7mol%-4HB) (Figure 9 B4) after 12-weeks implantation.

Molecular weight changes of the PHA nanofibers after implantation were dependent on their monomer composition (Table 2). After 4-weeks implantation, bioabsorption was the most pronounced for the P(3HB-*co*-97mol%-4HB) nanofiber with 47% loss of weight average molecular weight (*M_w_*). In contrast, the P(3HB) and P(3HB-*co*-5mol%-3HH) nanofibers showed no *M_w_* loss, while the P(3HB-*co*-7mol%-4HB) recorded only 6% of *M_w_* loss. The slower bioabsorption rate of the copolymers with higher 3HB content over the copolymer with low 3HB content also revealed the importance of sample crystallinity. Since P(3HB) has high isotactic stereoregularity, it readily yields highly crystalline material [33,34,35,36,37,38,39,48,52]. Thus, the higher the content of 3HB, the more crystalline the polymer and hence slower bioabsorption rate is observed. The results obtained after 12-weeks implantation revealed only 7% and 8% of *M_w_* loss for the P(3HB) and P(3HB-*co*-5mol%-3HH) nanofibers, respectively. Interestingly, the P(3HB-*co*-7mol%-4HB) nanofibers lost 43% of it *M_w_*. Possibly, the presence of thin nanofibers in the matrix of the electrospun P(3HB-*co*-7mol%-4HB) contributed to its relatively fast bioabsorption due to high surface area to volume ratio. Unexpectedly, the P(3HB-*co*-97mol%-4HB) nanofibers recorded only 37% of *M_w_* loss after 12 weeks. A possible reason for the low *M_w_* loss despite having high mass loss (as seen in Figure 7 whereby only a small piece of the scaffold was retrieved) is that the biodegraded products of lower molecular weight diffused away more easily due to increased scaffold porosity thus leaving only the parts of scaffold with higher molecular weight. Based on the results obtained after 4 weeks, the bioabsorption rate of the P(3HB-*co*-97mol%-4HB) nanofibers was found to be fast relative to the other two PHA copolymers.

**Table 2:** Molecular weight of PHA copolymers in the as-spun state, after sterilization, and after *in vivo* implantation and *in vivo* degradation [54].

Material	Condition	*M*_n_ x 10^5^	*M*_w_ / *M*_n_
P(3HB)	As-spun	3.5	3.1
	Sterilized	2.8	3.3
	4 weeks *in vivo*	4.6	2.6
	*in vitro*	4.2	2.6
	12 weeks *in vivo*	2.9	2.9
	*in vitro*	5.2	3.3
P(3HB-*co*-5mol%-3HH)	As-spun	3.6	3.6
	Sterilized	3.3	3.6
	4 weeks *in vivo*	3.9	3.1
	*in vitro*	2.6	4.3
	12 weeks *in vivo*	3.3	3.3
	*in vitro*	3.0	4.3
P(3HB-*co*-7mol%-4HB)	As-spun	2.3	3.0
	Sterilized	2.3	3.0
	4 weeks *in vivo*	2.5	2.6
	*in vitro*	2.3	2.6
	12 weeks *in vivo*	1.4	2.7
	*in vitro*	1.9	2.6
P(3HB-*co*-97mol%-4HB)	As-spun	1.1	1.5
	Sterilized	1.1	1.8
	4 weeks *in vivo*	0.6	1.6
	*in vitro*	1.3	2.0
	12 weeks *in vivo*	0.6	2.0
	*in vitro*	1.2	1.8

**Figure 10:**
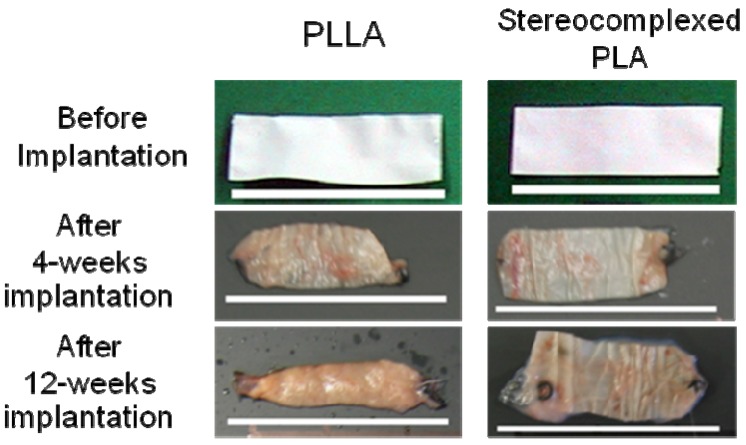
PLA nanofiber mats after subcutaneous implantation in rat for 4 to 12 weeks. Scale bars = 3cm [55].

## 4. Biocompatibility of PLA Nanofibers

Figure 10 shows the photographs of PLA nanofibers before and after 4-weeks and 12-weeks implantation, respectively. Significant reduction in the size of the PLLA nanofiber mat with increasing period of implantation was observed. In particular, the PLLA nanofiber mat after 12-weeks implantation was densely covered with the surrounding tissues and only small fragments of the nanofibers mat were recovered. On the other hand, the stereocomplexed PLA nanofiber mat showed a smaller degree of the reduction in size than the PLLA nanofiber mat. This suggests that the *in vivo* degradation of the stereocomplexed PLA nanofiber occurs slower than that of the PLLA nanofiber.

**Figure 11:**
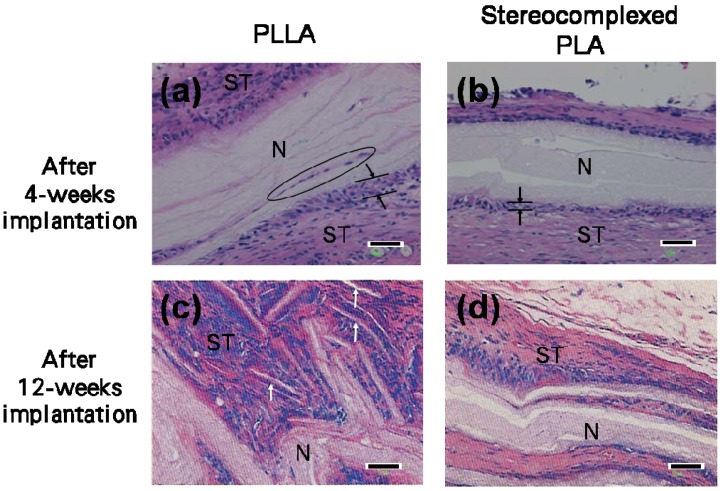
Phase contrast images of hematoxylin-eosin stained ultrathin sections of PLA nanofiber scaffolds after *in vivo* degradation for 4 to 12 weeks. N: Nanofiber scaffold. ST: Surrounding tissues. Scale bars = 50 μm [55].

Histological observations of the nanofibers were performed to investigate the degree of inflammatory reactions and penetration of the surrounding tissues into the nanofiber mats. Figure 11 shows the phase contrast images of ultrathin sections of the explanted nanofiber mats stained by hematoxylin-eosin. The nuclei of inflammatory cells were stained blue by the hematoxylin dye and their presence is an indication of tissue response towards the implanted nanofiber mats. As indicated by the arrows and lines in Figure 11(a), a thick layer of inflammatory cells was accumulated at the interface between the PLLA nanofiber mat and the surrounding tissues. In contrast, the layer of accumulated inflammatory cells was thinner for the stereocomplexed PLA nanofiber mat, as shown in Figure 11(b).

This indicates that the stereocomplexed PLA nanofiber causes a smaller degree of inflammatory reactions than the PLLA nanofiber. Furthermore, delamination [indicated by the ellipsoid in Figure 11(a)] occurred on the surface of the PLLA nanofiber mat and hence infiltration of the surrounding tissues was observed. However, no infiltration of the surrounding tissues was observed for the stereocomplexed PLA nanofiber mat. After 12-weeks implantation, while the PLLA nanofiber mat was significantly fragmented (white arrows indicate the fragmented nanofiber mat), the stereocomplexed PLA nanofibers retained the mat-like bulk morphology. These trends are well correlated with the bulk appearances of the nanofiber mats and support the observation that the *in vivo* degradation of the stereocomplexed PLA nanofiber proceeds slower than the PLLA nanofiber.

**Figure 12:**
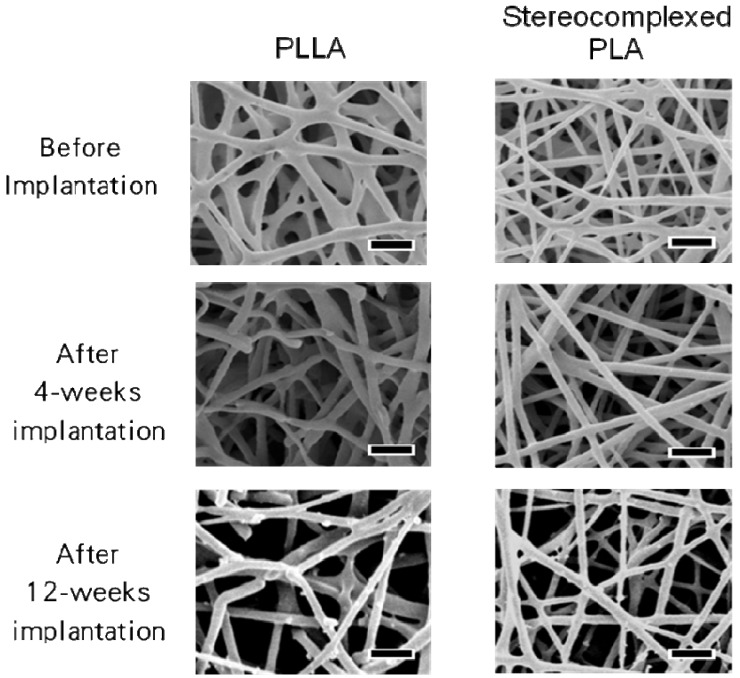
SEM images of PLA nanofiber scaffolds after subcutaneous implantation in rat for 4 to 12 weeks. Scale bars = 1 μm [55].

SEM observation was performed for the PLA nanofibers before and after 4-weeks and 12-weeks implantation. Figure 12 shows the SEM images of the PLA nanofibers before implantation, after implantation and incubation at different periods of time. As for PLLA, cleavage of each single nanofiber occurred after 4-weeks. Furthermore, after 12-weeks implantation, a decrease in the density of the nanofiber mat was observed. This is consistent with the histological image showing the fragmentation of the PLLA nanofiber mat. On the other hand, no cleavage of the stereocomplexed PLA nanofibers was observed, even after 12-weeks implantation.

**Figure 13:**
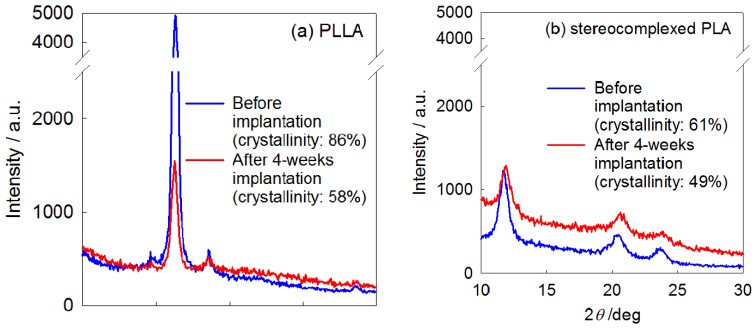
WAXD patterns of the PLLA and stereocomplexed PLA nanofibers before and after 4-weeks of implantation [55].

Figure 13 shows the WAXD patterns of the PLLA and stereocomplexed PLA nanofibers before and after 4-weeks implantation. While the PLLA nanofiber showed diffraction that are assigned to α-form crystal of PLA, the stereocomplexed PLA nanofiber showed diffractions assigned only to stereocomplexed crystal [17]. The crystallinity of both nanofibers was calculated as the ratio between the integrals of crystalline diffraction intensity and the total diffraction intensity. While the PLLA nanofiber showed considerable decrease in its crystallinity from 86% to 58%, the stereocomplexed PLA nanofiber showed a smaller decrease from 61% to 49%. These results show that the crystallinity of the stereocomplexed PLA is not so much lowered by implantation, while that of PLLA nanofiber significantly decreases. These results support the higher stability of stereocomplexed PLA nanofiber than PLLA nanofiber, as seen from visual inspection of the explanted nanofiber mat and the histological observation.

**Figure 14:**
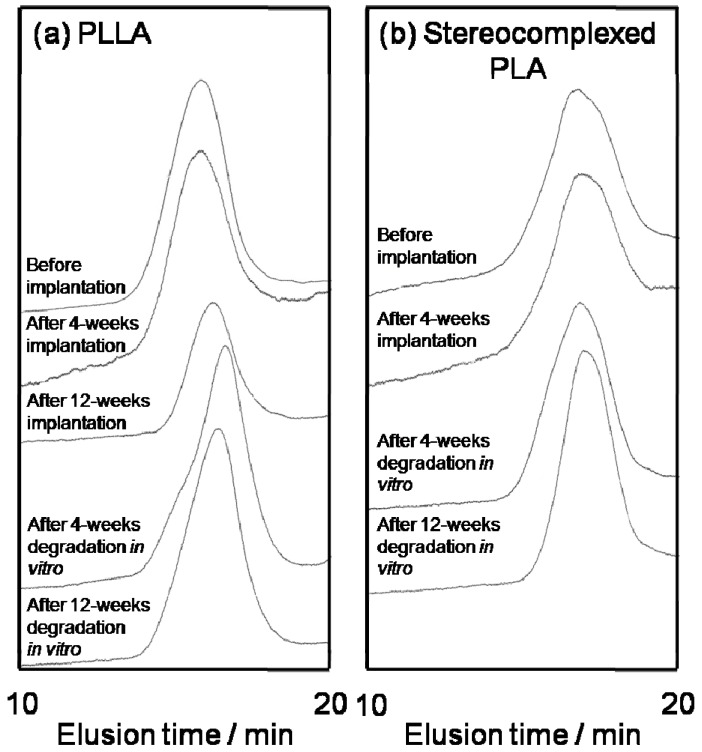
GPC elusion profiles of PLLA and stereocomplexed PLA nanofibers. **(a)** Before and after 4-weeks of implantation *in vivo*. **(b)** Before and after 4- and 12-week *in vitro* degradation [55].

The possibility of the cleavage of molecular chains during implantation, as suggested from SEM and WAXD data, was investigated by GPC analysis. The GPC elusion profiles are shown in Figure 14. Table 3 shows the number-averaged molecular weight, *M*_n_, and the polydispersity index, *M*_w_/*M*_n_, of the PLLA and stereocomplexed PLA nanofibers before and after 4-weeks implantation. The data for original PLLA are also shown in Table 3. In the case of 12-weeks, the GPC data of stereocomplexed PLA were not obtained because of its low solubility in chloroform. PLLA nanofiber showed a decrease in *M*_n_ during the implantation. In contrast, the *M*_n_ of stereocomplexed PLA nanofiber remained unchanged despite the decrease in *M*_w_/*M*_n_ for 4-weeks implantation. These results indicate that stereocomplexed PLA was not degraded during implantation, while the PLLA chains in the nanofibers were considerably degraded. Additionally, in the case of the stereocomplexed PLA nanofibers, the extraction of low molecular weight fraction might occur during implantation.

**Table 3:** Molecular weight of PLA nanofibers before and after 4-week and 12-week implantation *in vivo* [52].

	PLLA		PDLA		Stereocomplexed PLA	
	*M*_n_	*M*_w_/*M*_n_	*M*_n_	*M*_w_/*M*_n_	*M*_n_	*M*_w_/*M*_n_
Original	4.7 × 10^5^	1.8	2.2 × 10^5^	1.5	—	—
Nanofiber Before implantation	3.8 × 10^5^	2.3	—	—	8.7 × 10^4^	3.3
After 4-weeks implantation	3.0 × 10^5^	2.4	—	—	8.6 × 10^4^	2.3
After 12-weeks implantation	1.7 × 10^5^	2.3	—	—	—^a^	—^a^

^a^ Not obtained due to the poor solubility in chloroform.

**Figure 15:**
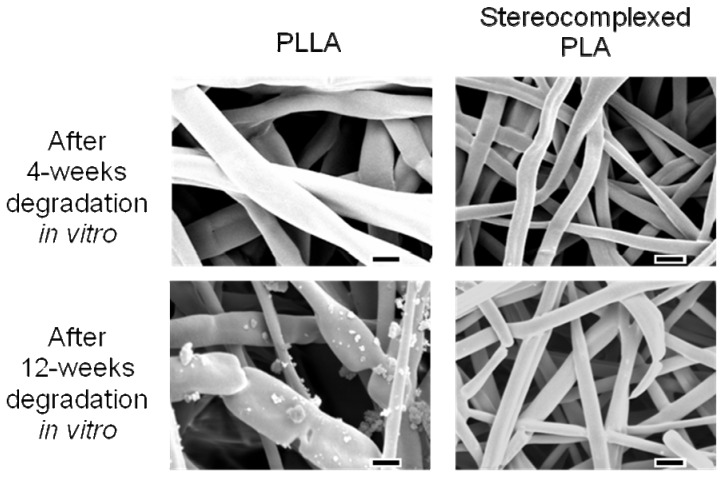
SEM images of PLLA (left) and stereocomplexed (right) nanofibers after 4-weeks (upper) and 12-weeks (lower) *in vitro* degradation in PBS. Scale bars = 1 μm [55].

In order to consider the results obtained from *in vivo* experiment in terms of biocompatibility and bioabsorption, changes in the structure and properties of the nanofibers after *in vitro* incubation was investigated. As seen in Figure 15, both PLLA and stereocomplexed PLA nanofibers after *in vitro* incubation showed a considerable increase in the fiber diameter. This suggests that the significant swelling of the nanofibers occurred during the incubation. Interestingly, the stereocomplexed PLA nanofiber showed smaller degree of swelling (from 300 nm to 600 nm) than the PLLA nanofiber (from 300 nm to 1,200 nm). Because strong interaction works between molecular chains of PLLA and PDLA in the stereocomplexed PLA nanofiber, the swelling of the stereocomplexed PLA nanofiber might be suppressed.

GPC data of the nanofibers before and after *in vitro* degradation were also obtained, as shown in Figure 14(b). The *M*_n_ and *M*_w_/*M*_n_ estimated from the GPC curves are listed in Table 4. The *M*_n_ of stereocomplexed PLA was almost unchanged while that of PLLA showed a decrease from 3.8 × 10^5^ to 1.8 × 10^5^. These trends are consistent with the molecular weight data before and after the implantation *in vivo* as shown in Table 1. The difference in the swelling behavior and molecular weight change *in vitro* between the stereocomplexed PLA and PLLA nanofibers may explain the results of the subcutaneous implantation *in vivo* in which the stereocomplexed PLA nanofiber showed smaller degree of absorption than the PLLA nanofibers.

**Table 4:** Molecular weight of PLA nanofibers before and after 4-week and 12-week degradation *in vitro* [55].

	PLLA		Stereocomplexed PLA	
	*M*_n_	*M*_w_/*M*_n_	*M*_n_	*M*_w_/*M*_n_
Nanofiber before implantation	3.8 × 10^5^	2.3	8.7 × 10^4^	3.3
After 4-weeks implantation	1.4 × 10^5^	3.5	8.4 × 10^4^	3.0
After 12-weeks implantation	1.8 × 10^5^	3.0	6.9 × 10^4^	2.2

## 5. Conclusions

The physiological responses of tissues against implanted foreign materials is one of the most significant topics to be considered in the development of medical biomaterials. In the case of polymeric biomaterials, the degree of the tissue responses, such as inflammatory reactions, partly depends on the chemical structure and, as a consequence, surface hydrophilic nature of the polymers [50]. Additionally, for biodegradable polymers, the degree of tissue responses is affected by the degradability *in vivo* [5]. For example, poly(glycolic acid) that undergoes degradation *in vivo* generally in 2-4 weeks is known to cause acute inflammatory reaction as the degradation proceeds. It is known that the hydrolysis by body fluids is the major mechanism contributing *in vivo* degradation of polymeric biomaterials.

We have shown in this review that the degradation behavior of PHAs *in vivo* is largely affected by the monomer composition [54]. Nanofiber scaffolds made from these PHAs, ranging from poly[(*R*)-3-hydroxybutyrate] to poly[(*R*)-3-hydroxybutyrate-*co*-97mol% 4-hydroxybutyrate] lead to contrasting tissue responses. The tissue responses were well correlated with the degradability of each polymer scaffolds. Furthermore, PHA nanofiber scaffolds with different monomer compositions showed variant mechanical properties, from flexible poly(3HB-*co*-97mol% 4HB) to rigid P(3HB) and P(3HB-*co*-5mol%-3HH). Such variety in the mechanical properties and degradability may facilitate the application of the PHA nanofibers for regeneration of organs or tissues with different mechanical properties.

The degradation study using nanofibers of PLLA and stereocomplexed PLA also suggested the correlation of the degree of inflammatory reaction *in vivo* with the change in the bulk size of each nanofiber mats. The changes in bulk size of the nanofibers were further correlated to the changes in the microscopic morphology, crystallinity, and molecular weight. All these factors give evidence that the stereocomplexed PLA nanofibers are more stable and thus provoke lower degree of inflammation *in vivo* than the PLLA nanofibers.

In general, inflammatory reaction is favored in the case where healing occurs in a short period of time. For example, inflammatory reaction stimulates and accelerates the regeneration of some kinds of epithelial tissues. On the other hand, when healing requires a longer time, chronic inflammatory response is not favored. For example, suppression of the inflammatory responses against artificial vessel has significance in the treatment of circulatory organs that requires a period of more than half a year. From this viewpoint, our results show that the stereocomplexed PLA nanofibers are suitable for the purposes where the chronic inflammatory reaction should be avoided, for example, guided nerve regeneration or blood vessel augmentation. On the other hand, conventional PLLA nanofibers may be suitable for the rapidly bioresorbable materials, for example, wound healing patches. Similarly, rigid P(3HB) or P(3HB-*co*-5mol%-3HH) nanofiber scaffold with low degradability may be suitable for the regeneration of hard tissues such as tendons or bones, while flexible and readily degradable P(3HB-*co*-97mol%-4HB) nanofiber may be suitable for the regeneration of soft tissues such as skins and bone marrows. Such versatility in the biodegradability of PLAs and PHAs would expand their potential as biomateirals. [54,55]

## References

[1] Langer R., Vacanti J.P. (1993). Tissue Engineering. Science.

[2] Gumbiner B.M. (1996). Cell Adhesion: The Molecular Basis of Tissue Architecture and Morphogenesis. Cell.

[3] Ma W., Tavakoli T., Derby E., Serebryakova Y., Rao M.S., Mattson M.P. (2008). Cell-Extracellular Matrix Interactions Regulate Neural Differentiation of Human Embryonic Stem Cells. BMC Dev. Biol..

[4] Griffith L.G. (2002). Emerging Design Principles in Biomaterials and Scaffolds for Tissue Engineering. Ann N. Y. Acad. Sci..

[5] Hasirci V., Lewandrowski K., Gresser J.D., Wise D.L., Trantolo D.J. (2001). Versatility of Biodegradable Biopolymers: Degradability and an *In Vivo* Application. J. Biotechnol..

[6] Doi Y. (1990). Fermentation and Analysis of Microbial Polyesters. Microbial Polyesters.

[7] Lemoigne M. (1926). Produits de déshydration et de polymérization de l’acide β=oxybutyrique. Bull. Soc. Chim. Biol..

[8] Lenz R.W., Marchessault R.H. (2005). Bacterial Polyesters: Biosynthesis, Biodegradable Plastics and Biotechnology. Biomacromolecules.

[9] Iwata T. (2005). Strong Fibers and Films of Microbial Polyesters. Macromol. Biosci..

[10] Martin D.P., Williams S.F. (2003). Medical Applications of Poly-4-hydroxybutyrate: A Strong Flexible Absorbable Biomaterial. Biochem. Eng. J..

[11] Hocking P.J., Marchessault R.H., Griffin G.J.L. (1994). Biopolyesters. Chemistry and Technology of Biodegradable Polymers.

[12] Williams S.F., Martin D.P., Doi Y., Steinbüchel A. (2002). Applications of PHAs in Medicine and Pharmacy. Biopolymers.

[13] Ikada Y., Tsuji H. (2000). Biodegradable Polyesters for Medical and Ecological Applications. Macromol. Rapid Commun..

[14] Iwata T., Doi Y. (2001). Alkaline Hydrolysis of Solution-Grown Poly(L-lactic acid) Single Crystals. Sen’i Gakkaishi.

[15] Ikada Y., Jamshidi K., Tsuji H., Hyon S.H. (1987). Stereocomplex Formation between Enantiomeric Poly(lactides). Macromolecules.

[16] Tsuji H. (2005). Poly(lactide) Stereocomplexes: Formation, Structure, Properties, Degradation, and Applications. Macromol. Biosci..

[17] Okihara T., Tsuji M., Kawaguchi A., Katayama K., Tsuji H., Hyon S.H., Ikada Y. (1991). Crystal Structure of Stereocomplex of Poly(l-lactide) and Poly(d-lactide). J. Macromol. Sci. -Phys..

[18] Tsuji H. (2000). *In Vitro* Hydrolysis of Blends from Enantiomeric Poly(lactide)s Part 1. Well-Stereo-Complexed Blend and Non-Blended Films. Polymer.

[19] Tsuji H., Suzuki M. (2001). *In Vitro* Hydrolysis of Enantiomeric Poly(lactide)s. 2. Well-Stereocomplexed Fiber and Film. Sen’I Gakkaishi.

[20] Tsuji H., Miyaushi S. (2001). Enzymatic Hydrolysis of Poly(lactide)s: Effects of Molecular Weight, L-Lactide Content, and Enantiomeric and Diastereoisomeric Polymer Blending. Biomacromolecules.

[21] Reneker D.H., Chun I. (1996). Nanometre Diameter Fibres of Polymer, Produced by Electrospinning. Nanotechnology.

[22] Morota K., Matsumoto K., Mizukoshi T., Konosu Y., Minagawa M., Tanioka A., Yamagata Y., Inoue K. (2004). Poly(ethylene oxide) Thin Films Produced by Electrospray Deposition: Morphology Control and Additive Effects of Alcohols on Nanostructure. J. Colloid Interface Sci..

[23] Murugan R., Ramakrishna S. (2006). Nano-Featured Scaffolds for Tissue Engineering: A Review of Spinning Methodologies. Tissue Eng..

[24] Reneker D.H., Yarin A.L., Fong H., Koombhongse S. (2000). Bending Instability of Electrically Charged Liquid Jets of Polymer Solutions in Electrospinning. J. Appl. Phys..

[25] Buchko C.J., Chen L.C., Shen Y., Martin D.C. (1999). Processing and Microstructural Characterization of Porous Biocompatible Protein Polymer Thin Films. Polymer.

[26] Zong X., Kim K., Fang D., Ran S., Hsiao B.S., Chu B. (2002). Structure and Process Relationship of Electrospun Bioabsorbable Nanofiber Membranes. Polymer.

[27] Zong X., Ran S., Kim K.-S., Fang D., Hsiao B., Chu B. (2003). Structure and Morphology Changes during *in Vitro* Degradation of Electrospun Poly(glycolide-co-lactide) Nanofiber Membrane. Biomacromolecules.

[28] Choi J.S., Lee S.W., Jeong L., Bae S.-H., Min B.C., Youk J.H., Park W.H. (2004). Effect of Organosoluble Salts on the Nanofibrous Structure of Electrospun Poly(3-hydroxybutyrate-*co*-3-hydroxyvalerate). Int. J. Biol. Macromol..

[29] Ito Y., Hasuda H., Kamitakahara M., Ohtsuki C., Tanihara M., Kang I.-K., Kwon O.H. (2005). A Composite of Hydroxyapatite with Electrospun Biodegradable Nanofibers as a Tissue Engineering Material. J. Biosci. Bioeng..

[30] Sombatmankhong K., Suwantong O., Waleetorncheepsawat S., Supaphol P. (2006). Electrospun Fiber Mats of Poly(3-hydroxybutyrate), Poly(3-hydroxybutyrate-co-3-hydroxyvalerate), and Their Blends. J. Polym. Sci., Part B: Polym. Phys..

[31] Kim K., Yu M., Zong X., Chiu J., Fang D., Seo Y.-S., Hsiao B.S., Chu B., Hadjiargyrou M. (2003). Control of Degradation Rate and Hydrophilicity in Electrospun Non-Woven Poly(d,l-lactide) Nanofiber Scaffolds for Biomedical Applications. Biomaterials.

[32] Zeng J., Chen X., Liang Q., Xu X., Jing X. (2004). Enzymatic Degradation of Poly(L-lactide) and Poly(*ε*-caprolactone) Electrospun Fibers. Macromol. Biosci..

[33] You Y., Min B.-M., Lee S.J., Lee T.S., Park W.H. (2005). *In Vitro* Degradation Behavior of Electrospun Polyglycolide, Polylactide, and Poly(lactide-*co*-glycolide). J. Appl. Polym. Sci..

[34] Orts W.J., Marchessault R.H., Bluhm T.L., Hamer G.K. (1990). Observation of Strain-Induced β-Form in Poly(β-hydroxyalkanoates). Macromolecules.

[35] Aoyagi Y., Doi Y., Iwata T. (2003). Mechanical Properties and Highly Ordered Structure of Ultra-High-Molecular-Weight Poly[(*R*)-3-hydroxybutyrate] Films: Effects of Annealing and Two-Step Drawing. Polym. Degrad. Stab..

[36] Iwata T., Tsunoda K., Aoyagi Y., Kusaka S., Yonezawa N., Doi Y. (2003). Mechanical Properties of Uniaxially Cold-Drawn Films of Poly[(*R*)-3-hydroxybutyrate]. Polym. Degrad. Stab..

[37] Iwata T., Aoyagi Y., Fujita M., Yamane H., Doi Y., Suzuki Y., Takeuchi A., Uesugi K. (2004). Processing of a Strong Biodegradable Poly[(*R*)-3-hydroxybutyrate] Fiber and a New Fiber Structure Revealed by Micro-Beam X-Ray Diffraction with Synchrotron Radiation. Macromol. Rapid Commun..

[38] Tanaka T., Fujita M., Takeuchi A., Suzuki Y., Uesugi K., Ito K., Fujisawa T., Doi Y., Iwata T. (2006). Formation of Highly Ordered Structure in Poly[(*R*)-3-hydroxybutyrate-*co*-(*R*)-3-hydroxyvalerate] Highstrength Fibers. Macromolecules.

[39] Iwata T., Fujita M., Aoyagi Y., Doi Y., Fujisawa T. (2005). Time-Resolved X-Ray Diffraction Study on Poly[(*R*)-3-hydroxybutyrate] Films during Two-Step-Drawing: Generation Mechanism of Planar Zigzag Structure. Biomacromolecules.

[40] Dong H., Nyame V., Macdiarmid A.G., Jones W.E. (2004). Polyaniline/Poly(methyl methacrylate) Coaxial Fibers: The Fabrication and Effects of the Solution Properties on the Morphology of Electrospun Core Fibers. J. Polym. Sci., Part B: Polym. Phys..

[41] Lyons J., Li C., Ko F. (2004). Melt-electrospinning Part I: Processing Parameters and Geometric Properties. Polymer.

[42] Fong H., Chun I., Reneker D.H. (1999). Beaded Nanofibers Formed during Electrospinning. Polymer.

[43] Zuo W., Zhu M., Yang W., Yu H., Chen Y., Zhang Y. (2005). Experimental Study on Relationship between Jet Stability and Formation of Beaded Fibers during Electrospinning. Polym. Eng. Sci..

[44] Tsuji H., Ikada Y., Hyon S.H., Kimura Y., Kitao T. (1994). Stereocomplex Formation between Enantiomeric Poly(lactic acid)s. VIII. Complex Fibers Spun from Mixed Solution of Poly(D-lactic acid) and poly(L-lactic acid). J. Appl. Polym. Sci..

[45] Takasaki M., Ito H., Kikutani T. (2003). Structure Development of Polylactides with Various D-Lactide Contents in the High-Speed Melt Spinning Process. J. Macromol. Sci., Part B: Phys..

[46] Furuhashi Y., Kimura Y., Yamane H. (2007). Higher Order Structural Analysis of Stereocomplex-Type Poly(lactic acid) Melt-Spun Fibers. J. Polym. Sci., Part B: Polym. Phys..

[47] Tsuji H., Nakano M., Hashimoto M., Takashima K., Katsura S., Mizuno A. (2006). Electrospinning of Poly(lactic acid) Stereocomplex Nanofibers. Biomacromolecules.

[48] Iwata T., Aoyagi Y., Tanaka T., Fujita M., Takeuchi A., Suzuki Y., Uesugi K. (2006). Micro-Beam X-Ray Diffraction and Enzymatic Degradation of Poly[(*R*)-3-hydroxybutyrate] Fibers with Two Kinds of Molecular Conformations. Macromolecules.

[49] Rappolee D.A., Mark D., Banda M.J., Werb Z. (1988). Wound Macrophages Express TGF-alpha and Other Growth Factors *in Vivo*: Analysis by mRNA Phenotyping. Science.

[50] Martin D.P., Skraly F.A., Williams S.F. (1999). Polyhydroxyalkanoate Compositions Having Controlled Degradation Rats. PCT Patent Application.

[51] Qu X.H., Wu Q., Zhang K.Y., Chen G.Q. (2006). *In Vivo* Studies of Poly(3-hydroxybutyrate-*co*-3-hydroxyhexanoate) Based Polymers: Biodegradation and Tissue Reactions. Biomaterials.

[52] Ishii D., Lee W.-K., Kasuya K., Iwata T. (2007). Fine Structure and Enzymatic Degradation of Poly[(*R*)-3-hydroxybutyrate] and Stereocomplexed Poly(lactide) Nanofibers. J. Biotechnol..

[53] Wang Y.-X., Robertson J.L., Spillman W.B., Claus R.O. (2004). Effects of the Chemical Structure and the Surface Properties of Polymeric Biomaterials on Their Biocompatibility. Pharm. Res..

[54] Tang H.Y., Ishii D., Mahara A., Murakami S., Yamaoka T., Sudesh K., Samian R., Fujita M., Maeda M., Iwata T. (2008). Scaffolds from Electrospun Polyhydroxyalkanoate Copolymers: Fabrication, Characterization, Bioabsorption and Tissue Response. Biomaterials.

[55] Ishii D., Tang H. Y., Mahara A., Murakami S., Yamaoka T., Iwata T. (2009). *In Vivo* Tissue Response and Degradation Behavior of PLLA and Stereocomplexed PLA Nanofibers. Biomacromolecules.

[56] Zong X., Bien H., Chung C.-Y., Yin L., Fang D., Hsiao B.S., Chu B., Entcheva E. (2005). Electrospun Fine-Textured Scaffolds for heart Tissue Constructs. Biomaterials.

